# Size Dependent Cellular Uptake of Rod-like Bionanoparticles with Different Aspect Ratios

**DOI:** 10.1038/srep24567

**Published:** 2016-04-15

**Authors:** Xiangxiang Liu, Fengchi Wu, Ye Tian, Man Wu, Quan Zhou, Shidong Jiang, Zhongwei Niu

**Affiliations:** 1 Key Laboratory of Photochemical Conversion and Optoelectronic Materials, Technical Institute of Physics and Chemistry, Chinese Academy of Sciences, Beijing 100190, China; 2 University of Chinese Academy of Sciences, Beijing 100049, China

## Abstract

Understanding the cellular internalization mechanism of nanoparticles is essential to study their biological fate. Especially, due to the anisotropic properties, rod-like nanoparticles have attracted growing interest for the enhanced internalization efficiency with respect to spherical nanoparticles. Here, to elucidate the effect of aspect ratio of rod-like nanoparticles on cellular uptake, tobacco mosaic virus (TMV), a typical rod-like bionanoparticle, is developed as a model. Nanorods with different aspect ratios can be obtained by ultrasound treatment and sucrose density gradient centrifugation. By incubating with epithelial and endothelial cells, we found that the rod-like bionanoparticles with various aspect ratios had different internalization pathways in different cell lines: microtubules transport in HeLa and clathrin-mediated uptake in HUVEC for TMV_4_ and TMV_8_; caveolae-mediated pathway and microtubules transport in HeLa and HUVEC for TMV_17_. Differently from most nanoparticles, for all the three TMV nano-rods with different aspect ratios, macropinocytosis takes no effect on the internalization in both cell types. This work provides a fundamental understanding of the influence of aspect ratio on cellular uptake decoupled from charge and material composition.

Cellular internalization pathway is essential to the understanding of viral infection and the development of therapeutic agents. Different viruses have specific pathways entering into cells. Take an example, Ebola virus enters into host cells through two-pore channels^1^. While for the synthetic cellular delivery nanoparticles, the pathway of their internalization dramatical influences the therapy efficiency. Recent studies have shown that shape, size^2^,^3^, surface properties^4^, rigidity^5^,^6^ and composition^7^ of nanoparticles have great impact on the cellular internalization and intracellular trafficking^8^. In particular, rod-like nanoparticles aroused lots of attention because of their dramaticaly difference from spherical nanoparticles in inherent chemical, electrical, magnetic, and optical anisotropy^9^,^10^,^11^. Unlike spherical nanoparticles, theoretically, rod-like nanoparticles enter into cells either by lying-down or standing-up manner^12^. Aspect ratio, as an important physical indicator for rod-like nanoparticles, has great impacts on cellular internalization efficiency and mechanism^13^, the formation of filopodia and the assembly behavior of actin cytoskeleton^14^. However, the understanding of the specific pathway entering into cells of rod-like particles with different aspect ratios still remains a challenge. This is mainly because for most of synthetic rod-like nanoparticles, such as gold^2^,^3^, silica^15^, and polymeric particles^16^,^17^ etc., the uniformity is hard to control during their synthesis processing. To dig out the cellular internalization mechanism, particles with highly monodisperse, identical surface properties and material compositions are needed^17^.

Biological macromolecules, however, are encoded by genes, which makes them definitely exhibit high uniformity in morphologies^18^. Plant viruses are favored by researchers gradually and used for biomedical applications^19^,^20^ for the advantages of the low production costs, high yields and nonpathogenic to animals. Tobacco mosaic virus (TMV), as an anisotropic rod-like biological entity with a contour length of 300 nm, an exterior diameter of 18 nm and interior diameter of 4 nm, was always used as a model shape^21^,^22^. It consists of 2130 identity proteins arranged helically around a single-stranded RNA. Besides the great advantage of morphological uniformity and easy functionalization, TMV offers the attractive properties of biocompatibility^23^,^24^, structural rigidity as well as chemical stability in biological environment. Furthermore, TMV has been used as scaffolds or biotemplates^25^,^26^,^27^,^28^ for nanotechnology applications^29^ due to its prevalent model of assembly^30^ based on that distinct RNA scaffolds can govern the assembly of TMV^31^,^32^,^33^ to realize precise control of nano-scale particles.

Based on these concerns, we report the study of the effect of aspect ratio on cellular uptake mechanism of rod-like bionanoparticles in epithelial and endothelial cells. As shown in Fig. 1, TMV can be broken into short rods by ultrasonic treatment. With this method, these short rods can still maintain their original rod morphology, exactly the same diameter, identical surface property and material compositions. Short rods with different aspect ratios can be separated by sucrose density gradient centrifugation separation. These bionanoparticles with different aspect ratios were then used to study their specific uptake mechanism in both epithelial and endothelial cells.

## Results and Discussion

**Figure 2:**
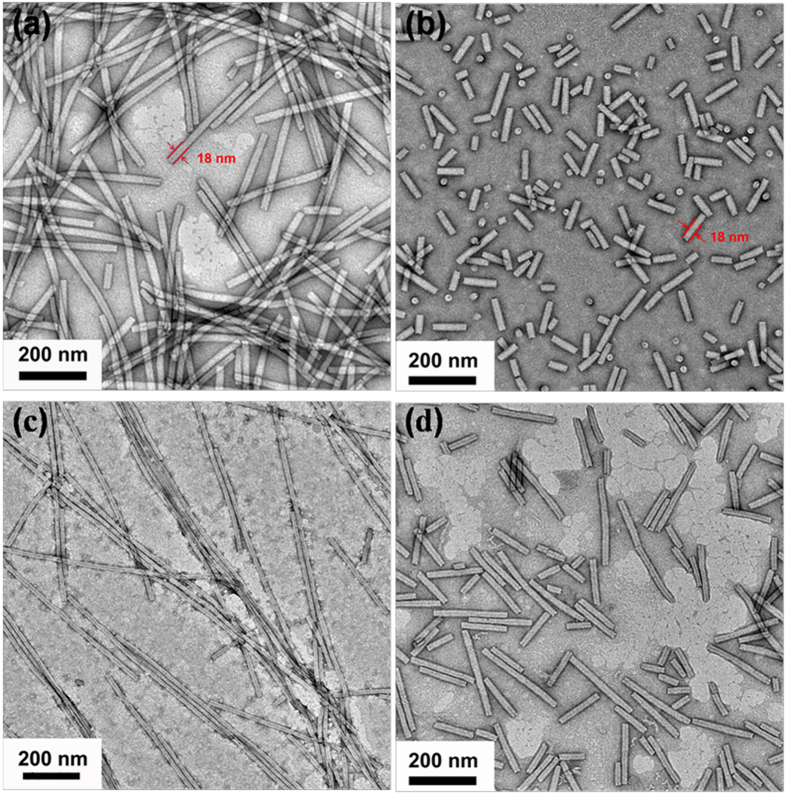
TEM images of TMV. The top row shows the morphology of native TMV (**a**) and usTMV (**b**). The next row indicates the self-assembling performance of usTMV: the usTMV can reassemble into long rods (**c**) and disassemble into short rods again (**d**) and the process is reversal between (**c**) and (**d**). The assembly and disassembly behavior occurs in pH 5.5 10 mM acetate buffer and pH 8.0 10 mM phosphate buffer, respectively.

Ultrasound could generate acoustic cavitation in liquids, which contains the formation, growth, and implosive collapse of bubbles. These processes produce extremely high temperatures and pressures in a microscopic region, thus trigger reactivity^34^. In another hand, viruses are generated by the nature own design with the protein assembly by the weak interaction. Thus, the mechanical force generated by the ultrasonication could easily destroy the integrity of TMV. In a typical experiment, a 5 mg/mL TMV in 0.01 M pH 8.0 phosphate buffer was treated with ultrasound in the ice bath for 30 minutes with three times. After treatment, the sample was directly characterized by the transmission electron microscopy (TEM). As shown in Fig. 2b, comparing to the TMV with the length 300 nm (Fig. 2a), after ultrasonication, short rods with the length 20~200 nm (coded as usTMV) are visualized. The diameter of short rods still remains 18 nm, which is the same as native TMV.

One critical consideration is the structural stability of TMV after ultrasonic treatment. Sonication could denature proteins through large pressure, temperature gradients, high shear forces and generating free radicals^35^,^36^. However, the suitable ultrasound parameter, such as lower ultrasound power and shorter irradiation time, could minimally damage protein^37^. Based on that, we chose thrice 30 min ultrasonic treatment in ice bath. In our research, the main focus is the aspect ratio-dependent internalization mechanism of rod-like nanoparticles, which have the monodispersed diameter and behave the same physiochemical nature. Whether the TMV short rods still have bio-activity after ultrasonication treatment is very important. TMV coat proteins have the capacity to self-assemble into well-defined nano-rods or nano-disks at specific pH value and ionic strength^38^,^39^. So we investigate the bio-activity of TMV short rods through their assembling behavior. As shown in Fig. 2c,d, ultrasonic treatments have nearly no effect on the self-assembly performance of TMV short rods: short rods can still reassemble into long rods in pH 5.5 acetate buffer, subsequently disassemble into short rods in pH 8.0 phosphate buffer. Furthermore, the assembly and disassembly behavior of TMV short rods is a reversal process. Based on above analysis, ultrasonication did not alter the same bioactivity of short rods and long rods.

Apart from the structural stability, another important item is if the surface charge of TMV short rods is changed after ultrasonication, which is one of the factors affecting cellular internalization. We measured the zeta potentials of TMV in PBS before and after ultrasonication (Table 1), and the results showed that ultrasonication did not change the surface charge in pH 7.4 (physiological pH) and pH 8.0 (experimental pH) PBS. Since the shape, bioactivity and surface charge are steady, we can treat this work as merely size-dependent study.

**Figure 3:**
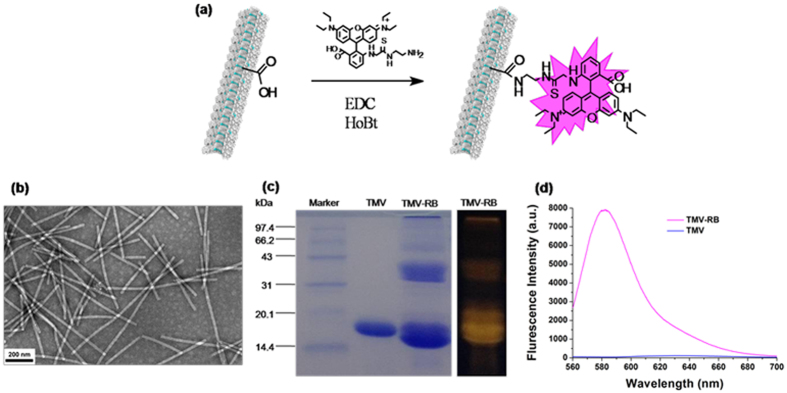
Schematic diagram and characterization of TMV labeled with RBITC-EDA (TMV-RB). (**a**) Modification of TMV in cavity with RBITC-EDA. (**b**) TEM image of TMV-RB confirms the integrity of TMV after modification. (**c**) SDS-PAGE visualized by Coomassie staining (left) and fluorescence (right). (**d**) Fluorescence spectra of TMV-RB and TMV.

To maintain the outer surface properties, we labeled TMV with Rhodamine B (RB) onto its inner cavity through one step reaction between amino groups of RB and two carboxylic groups of TMV to render it fluorescence detectable in the cellular uptake experiments (Fig. 3a). After reaction, the TEM showed that TMV still kept intact (Fig. 3b). The SDS-polyacrylamide gel electrophoresis (SDS-PAGE) demonstrated that TMV was successfully labeled by RB (Fig. 3c): the existence of red emission bands meant that RB was chemically conjugated onto TMV inner cavity. The number of RB labels per virion was calculated based on the UV-Vis spectroscopy. Through absorbance at 540 nm to confirm RB concentration, together with the Modified Lowry Protein Assay Kit to determine the exact TMV concentration, the conjugation density of RB was calculated as 0.4 RB molecules per TMV coat protein (852 dye molecules per TMV particle). Fluorescence spectra turned out that the conjugated RB provided TMV remarkable fluorescence emission (Fig. 3d).

**Figure 4:**
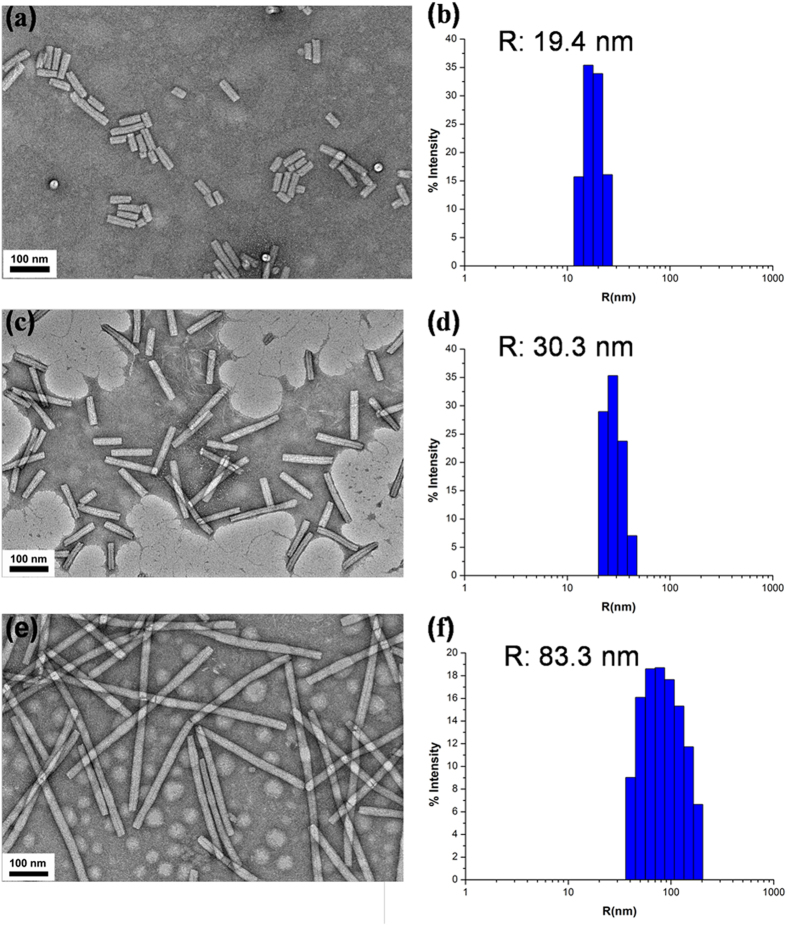
TEM images (a,c,e) and DLS (b,d,f) for TMV_4_ (a,b), TMV_8_ (c,d) and TMV_17_ (e,f).

After labeling, RB-TMV was treated with ultrasonication in the same condition as described before. The mixture of short rods was then separated by the sucrose density gradient centrifugation technique. This technique has been proved to be an efficient method to separate nanorods with different aspect ratios. As nanorods with different lengths have different sedimentation rates, giving rise to a broad band in the centrifuge tube under the centrifugal velocity, we collected the fraction from different part of the centrifuge tube: the upper part was the rods with short aspect ratios; the lower part was the rods with long aspect ratios. These rods were characterized by TEM and dynamic laser scattering (DLS) measurements. Comparing to RB-TMV with length 300 nm (Fig. 4e) and a hydrodynamic diameter of 80.3 nm, the short rods with average length about 70 nm (Fig. 4a) can be clearly visualized. DLS measurement shows the short rods have a narrow size distribution with hydrodynamic diameter of 19.4 nm (Fig. 4b). Collected from the lower part, the rods with average length about 140 nm (Fig. 4c) and narrow size distribution (measured by DLS, Fig. 4d) were obtained.

**Figure 5:**
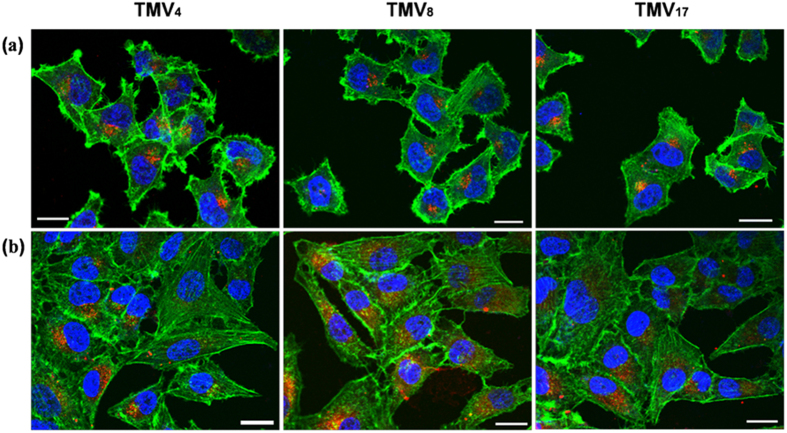
CLSM images of cellular uptake and distribution of TMV_4_, TMV_8_, and TMV_17_ in (a) HeLa cells and (b) HUVEC cells after co-incubation for 4 hours with TMV. Green color collected from 500–550 nm show F-actin stained by Alexa Fluore-488 phalloidin. Cell nucleus were stained by To-pro-3 (collected from 662–737 nm), and the color was changed to blue by the CLSM software. RB was shown in red color (collected from 570–610 nm), indicating the distribution of TMV rods. The scale bars in figures represent 20 μm.

**Figure 6:**
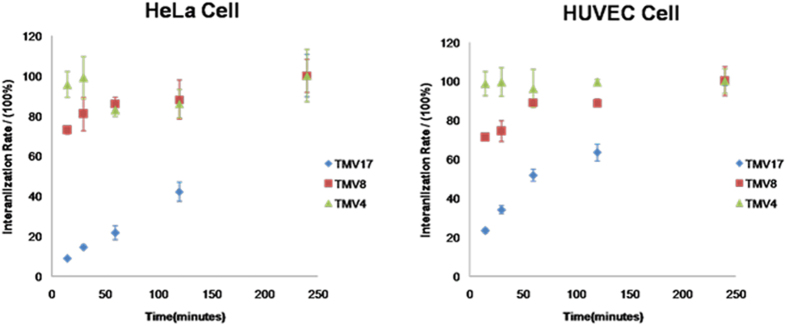
The kinetics of internalization of nanorod-like TMV with HeLa and HEVEC cells over a 4 h incubation period at 37 °C. Cells incubated with TMV for 4 hours were regarded as 100%. The error bars are the standard deviation from three independent groups.

By co-culturing as-prepared RB labeled TMV rods with different cell lines for determined time, the cells were visualized on confocal laser scanning microscopy (CLSM). Wege and Steinmetz group recently reported that the behavior of TMV, RGD-labeled TMV and PEGylated TMV in epithelial tumor cells (HT-29 cells) and immune cells significantly rely on the aspect ratio^32^. However, the internalization mechanisms are not emphasized. For most nanoparticles, the transendothelial and transepithelial pathway are different, because of the difference in membrane proteins, permeability, cell polarity and membrane asymmetry between endothelial and epithelial cells. For example, the asymmetry of the cell membrane is more prominent in most epithelial cells; the caveolae expression on endothelial cell membrane is higher than that on epithelial cell membrane. So in our study, an epithelial cell line (HeLa cell line) and an endothelial cell line (human umbilical vein endothelial cell (HUVEC)) were used to investigate the endocytic processes of the TMV rods with different aspect ratios. As shown in Fig. 5, CLSM images showed that all the samples could be endocytosed into Hela cells and HUVEC cells, accumulating around cell nuclei. To evaluate the kinetics of particle internalization, we applied flow cytometry and studied the time course of cellular uptake from 15 min to 4 h (Fig. 6). Computer simulations have shown that cell membrane wrapping of anisotropic particles is intricate interplay between shape, initial orientation, surface adhesive energy, volume and aspect ratio^40^,^41^,^42^,^43^,^44^,^45^,^46^. Our data showed that the cellular internalization presented apparent dependence on aspect ratios of TMV. The internalization rate of TMV_4_ and TMV_8_ displayed cell type independent, and was obviously higher than TMV_17_. Shorter ones were found to undergo endocytosis easier than longer ones. The reason may be that the larger volume, increased surface area and high aspect ratio are unfavorable for complete membrane wrapping^40^,^41^. Especially, the uptake rate of TMV_4_ was as high as 90% during the first 15 minutes. This high internalization efficiency of particles with aspect ratio of around 4 is in agreement with recent literature^9^,^13^. For the high-aspect-ratio TMV_17_, its internalization process was much slower, and its internalization kinetics exhibited different in various cell types: TMV_17_ was ingested faster in HUVEC than in HeLa cells.

**Figure 7:**
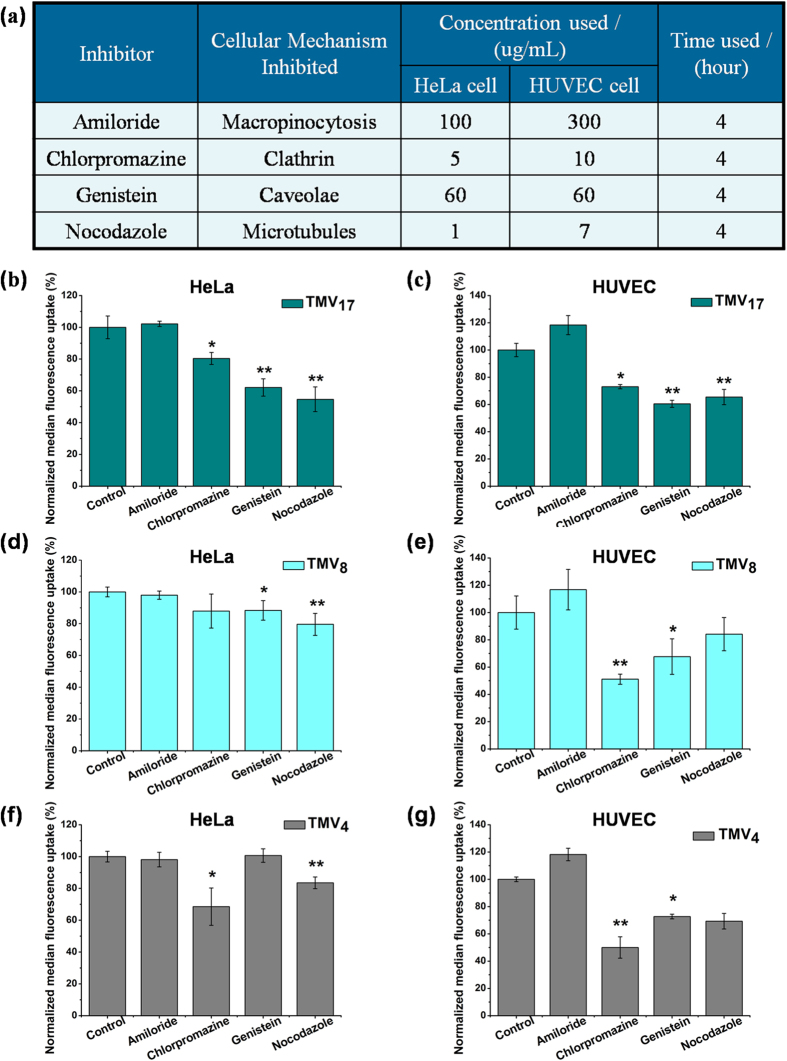
Effect of pharmacological inhibitors on uptake efficiency of rod-like TMV with various aspect ratios. (**a**) The inhibitors, functions, concentrations, and time we used for the internalization experiments. **(b,d,f**) Changes in normalized median fluorescence uptake of TMV_17_ (**b**) TMV_8_ (**d**) and TMV_4_ (**f**) due to pretreating with inhibitors in HeLa cells. (**c,e,g**) Changes in normalized median fluorescence uptake of TMV_17_ (**c**) TMV_8_ (**e**) and TMV_4_ (**g**) due to pretreating with inhibitors in HUVEC cells. All conditions were done in triplicates independently. Cells without inhibitor pretreating were taken as the control groups (set as 100% uptake efficiency). The error bars are the standard deviation from the mean. *p ≤ 0.05 and **p ≤ 0.01 compared with control groups.

Furthermore, we choose the widely applied pharmacological inhibitors to confirm the occurrence of macropinocytosis, microtubules transport, clathrin-mediated endocytosis and caveolae-mediated endocytosis^47^,^48^. The inhibitory functions of the pharmacological inhibitors^17^,^49^ and concentration used are listed in Fig. 7a. At the chosen concentration in Fig. 7a, the cells can achieve minimum 90% viability during 7 h co-incubation. The uptake efficiency of TMV_4_, TMV_8_ and TMV_17_ in HeLa cells and HUVEC cells were quantified by flow cytometry, shown in Fig. 7b–g. Amiloride blocks macropinocytosis, which is a non-specific pathway and is involved in most cell types. While for all the three TMV nano-rods with different aspect ratios, amiloride showed no inhibiting effect on both epithelial cell type (HeLa) and endothelial cell type (HUVEC). Clathrin-mediated endocytosis (blocked by chlorpromazine) is a best-characterized receptor-mediated pathway. It shows that it is a major internalization mechanism for both TMV_4_ and TMV_8_ in HUVEC cell line (about 50% decreasing, p < 0.01), and for all the three TMV rods, it plays a more important part in HUVEC cell line than in HeLa cell line. Caveolae, blocked by genistein, are a special type of lipid raft in many cell types, especially in endothelial cells. In Fig. 7, it shows that the caveolae-mediated mechanism behaves as a major internalization mechanism for all the three TMV rods in HUVEC cell line, and plays an important part for TMV_17_ in HeLa cell. Nocodazole is a pharmacological inhibitor for microtubule polymerization, which is responsible for a variety of cell movements, including the intracellular transport and positioning of membrane vesicles and organelles. Figure 7 showed that it could reduce the uptake efficiency of TMV_17_ to about 60% (p < 0.01) in both HeLa and HUVEC cell lines, and it was a significant inhibitor for TMV_4_ and TMV_8_ in HeLa cell line (p < 0.01).

Overall, the internalization mechanism of TMV nano-rods in both HeLa and HUVEC cells shows size-dependent. In HeLa cells (epithelial cell type), the prominent uptake mechanisms are caveolae-mediated endocytosis and microtubules transport for TMV_17_, microtubules transport for TMV_8_, and clathrin-mediated endocytosis and microtubules transport for TMV_4_. In HUVEC cells (endothelial cell type), the major internalization mechanisms are caveolae-mediated endocytosis and microtubules transport for TMV_17_, clathrin-mediated endocytosis and caveolae-mediated endocytosis for both TMV_8_ and TMV_4_.

## Conclusion

Bionanoparticle can be a great platform to act as a typical model to study the size or morphology dependent cellular internalization pathways. In this work we have shown that TMV based bionanorods with different aspect ratios have different pathways entering into different cells. Microtubules transport was mainly used in HeLa and clathrin-mediated uptake was mainly used in HUVEC for both TMV_4_ and TMV_8_, while caveolae-mediated and microtubules transports were predominantly used in HeLa and HUVEC for TMV_17_. Specifically, we have shown that none of these protein nanorods enters into different cells by macropinocytosis, which is dramatically different with synthetic nanorods. Additionally, the kinetic studies have shown that TMV short rods with aspect ratio of 4 and 8 are ingested much faster by both cells than TMV with the aspect ratio of 17. Our studies may have profound significance for the design of bionanoparticles based drug or gene carrier system.

## Experimental Section

### Materials and Chemicals

All reagents were used as received from commercial sources. Rhodamine B isothiocyanate (RBITC) and all the pharmacological inhibitions were purchased from Sigma. Ethylenediamine (EDA) and N-(3-Dimethylaminopropyl)-N’-ethylcarb odiimide (EDC) were obtained from Aladdin. 1-Hydroxybenzotriazole (HoBt) was purchased from J & K Chemical. D-(+)-Sucrose was obtained from TCI. Dulbecco’s modified Eagle’s medium (DMEM), fetal bovine serum (FBS), penicillin, streptomycin, Alexa Fluore-488 phalloidin molecular probe, To-PRO-3 iodide molecular probe, and Pro Long-Gold antifade reagent were purchased from Gibco (Life Technologies, Invitrogen, USA). Milli-Q water was used for all experiments.

### Synthesis of RBITC-EDA

Add 2 mL 39 mg/mL Rhodamine B isothiocyanate/DMF solution into 2 mL 7.3 mg/mL EDA/DMF solution dropwise with stirring, then stir for 20 h in dark at room temperature. The solvent was evaporated by rotator distillation under reduced pressure. The residue was dissolved in CH_2_Cl_2_ and precipitated into diethyl ether. The precipitation was dried to constant weight in vacuum.

### Labeling TMV with RBITC-EDA

TMV was extracted from infected leaves using established method^23^,^50^. The concentration of TMV was determined by Lowry Protein Assay kit (Solarbio Science & Technology Co., Ltd). A solution of 2 mg/ml TMV in HEPES buffer (pH 8.0, 10 mM) was added to the round-bottle flask with gentle vortexing. Then add 25 eq HoBt and 5 eq EDC to the flask successively every 30 min to activate the carboxyl groups in the inner cavity of TMV. Subsequently, put 5 eq RBITC-EDA dropwise into the flask. The mixture was stirred in dark at 4 °C for 18 h, during which add 5 eq EDC twice to the reaction system. Samples need to dialysis against PBS buffer (pH 8.0, 10 mM) using a Biotech CE Dialysis Membranes (Spectrum Laboratories, Inc., MWCO 1000 kD) in dark for 48 h with changes of buffer. Fluorescence spectra of the labeled TMV were recorded on a Hitachi F-4600 fluorescence spectrophotometer.

### Ultrasonic Treatment

Ultrasonic Cell Disruption System (model JY92-IIN, SCIENTZ) was used for ultrasonic treatment. Sample solutions were sonicated with a set of 10% electric power, 0.5 duty factors (5 s pulses on and 5 s pulses off), and irradiation time of 30 min. A 5 ml TMV in PBS buffer (pH 8.0, 10 mM) in each treatment was poured into the polystyrene tube immersed in the ice bath. Each treatment was replicated three times.

Zeta potentials of TMV and usTMV were measured by Zetasizer nano ZSP (Malvern).

### Separation of TMV with Different aspect ratios

Stepwise gradients were prepared in 13 ml tubes by layering 2 ml volumes of 33% (wt/v), 30%, 27%, 24%, and 21% sucrose solutions diluted in PBS (pH 8.0, 10 mM) from bottom to top layer. The sucrose solutions were stored at 4 °C before using. Immediately, 0.2 ml usTMV in PBS was layered on top of the gradient. The tubes were centrifuged at 30,000 rpm for 2.5 h at 4 °C using a Beckman SW40 rotor. Each fraction (1 ml) was collected from top to down. The third fraction was collected as the short aspect ratio of TMV fraction, and the sixth fraction was collected as the medium aspect ratio of TMV fraction. While for the separation of long aspect ratio of TMV, the sucrose gradients were 70% (wt/v), 60%, 50%, 40%, and 30% sucrose in PBS from bottom to top, and 0.2 ml TMV was put on the top layer, then the same ultracentrifugation protocol was used. The sucrose layer with sample was collected as the long aspect ratio of TMV.

After centrifugation, the TMV was purified from sucrose by dialysis against pH 8.0 PBS (10 mM) buffer in dark at 4 °C over 6 cycles (6 + hours each time) and condensed using a Microcon (Millipore) spin filter unit (MWCO 10 kD & 100 kD).

The hydrodynamic diameter of TMV with different aspect ratios in PBS was determined by Dynamic Light Scattering (DLS) on a DynaPro NanoStar instrument (Wyatt Technology) with a He-Ne laser (λ = 659 nm) operated at 10 mW and analyzed with Dynamics software v6.11. Experiments were run at 25 °C in triplicate, with 10 acquisitions per measurement.

The visualization of TMV was performed with a transmission electron microscopy (TEM, JEM-2100F JEOL, Japan) with an accelerating voltage of 120 kV and the samples were stained by 0.2% uranium acetate before observation.

### Cell Culture

HeLa (human cervical cancer epithelial cell) and HUVEC (human umbilical vein endothelial cell) were incubated in DMEM medium with 1% antibiotics (penicillin-streptomycin) and 10% FBS at 37 °C in a humidified atmosphere containing 5% CO_2_.

### Co-incubation of TMV with various aspect ratios with cells

HeLa epithelial cells and HUVEC endothelial cells were seeded respectively onto glass coverslips in a 6 well culture plate overnight. Thereafter, the TMV with various aspect ratios dispersed in the culture medium and the cells were incubated for another 4 h. After removing the medium and washing thrice with PBS, the cells were fixed for 10 min with 4% paraformaldehyde in PBS at room temperature, and washed thrice with PBS. Then the cells were permeablized with 0.1% Triton X-100 in PBS for 5 min at room temperature, and washed thrice with PBS. F-actin was then stained with Alexa Fluore-488 phalloidin in 1% BSA/PBS for 15 min at room temperature, washed thrice with PBS and the nucleus was stained with To-PRO-3 iodide in PBS (10 ug/ml RNase/PBS) for 20 min at room temperature. Coverslips were washed thrice with PBS and water, and sealed using Pro Long-Gold antifade reagent. Cells were observed by Nikon Eclipse Ti confocal laser scanning microscopy (CLSM).

### Pharmacological Inhibition Studies by Flow Cytometry

For the performance of flow cytometry, HeLa epithelial cells and HUVEC endothelial cells were seeded (50,000 cells per well) in a 24 well tissue culture plate and then allowed to adhere overnight. Pharmacological inhibitors were used and added to cells at the concentration we used before^30^ described in Table 1. Cells were incubated for 1 h after which TMV with various aspect ratios were added to cells (10 ug/ml) for another 4 h. Then cells were washed thrice with PBS, trypsinized and collected into 10% FACS buffer (10% FBS in PBS), and analyzed using a flow cytometry (BD Calibur) and 10,000 gated events were recorded. The untreated cells were taken for background fluorescence, which was subtracted from test samples. For each sample (TMV_17_, TMV_8_ or TMV_4_), cells without inhibitor pretreating were taken as the control group (set as the 100% uptake efficiency). Data show average and standard deviation from three independent experiments.

### Kinetics of TMV internalization

The HeLa and HUVEC cell lines were used to investigate the uptake rate of TMV with various aspect ratios. Samples were incubated with cells over a time course ranging from 15 min to 4 h (37 °C, 5% CO2). After cell/particle incubation, the cells were washed and detached by trypsinization. Cells were then collected in PBS with 10% FBS, and samples were analyzed by flow cytometry. There were 10,000 cells measured in each sample.

## Additional Information

**How to cite this article**: Liu, X. *et al.* Size Dependent Cellular Uptake of Rod-like Bionanoparticles with Different Aspect Ratios. *Sci. Rep.* **6**, 24567; doi: 10.1038/srep24567 (2016).

## Figures and Tables

**Figure 1:**
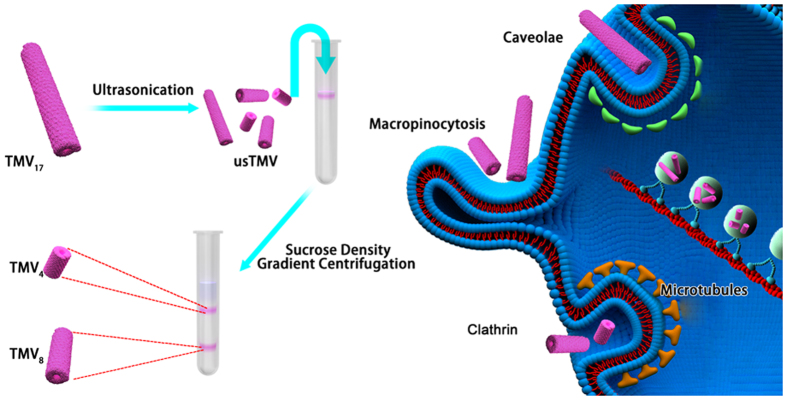
Schematic illustration of preparation of bionanoparitcles with different aspect ratios and their internalization mechanisms. TMV_17_: native TMV with aspect ratio of 17; TMV_8_: short TMV rods with aspect ratio of 8; TMV_4_: short TMV rods with aspect ratio of 4.

**Table 1:** Zeta potentials of TMV before and after ultrasonication.

Buffer	Sample	Zeta Potential/ (mV)
pH 7.4 10 mM PBS	TMV	−36.3 ± 2.68
	usTMV	−36.5 ± 2.65
pH 8.0 10 mM PBS	TMV	−39.3 ± 2.00
	usTMV	−39.8 ± 2.82
